# Altered Effective Connectivity Measured by Resting-State Functional Magnetic Resonance Imaging in Posterior Parietal-Frontal-Striatum Circuit in Patients With Disorder of Consciousness

**DOI:** 10.3389/fnins.2021.766633

**Published:** 2022-01-20

**Authors:** Linglong Chen, Bo Rao, Sirui Li, Lei Gao, Yu Xie, Xuan Dai, Kai Fu, Xu Zhi Peng, Haibo Xu

**Affiliations:** ^1^Department of Radiology, Zhongnan Hospital of Wuhan University, Wuhan, China; ^2^Department of Neurology, Zhongnan Hospital of Wuhan University, Wuhan, China; ^3^Department of Neurosurgery, Zhongnan Hospital of Wuhan University, Wuhan, China

**Keywords:** disorders of consciousness, seed-based *d* mapping permutation of subject images, spectral dynamic causal modeling, corticostriatal connection, preCUN/PCC

## Abstract

**Objective:**

Disorder of consciousness (DoC) resulting from severe brain injury is characterized by cortical and subcortical dysconnectivity. However, research on seed-based effective connectivity (EC) of DoC might be questioned as to the heterogeneity of prior assumptions.

**Methods:**

Functional MRI data of 16 DoC patients and 16 demographically matched healthy individuals were analyzed. Revised coma recovery scale (CRS-R) scores of patients were acquired. Seed-based *d* mapping permutation of subject images (SDM-PSI) of meta-analysis was performed to quantitatively synthesize results from neuroimaging studies that evaluated resting-state functional activity in DoC patients. Spectral dynamic causal modeling (spDCM) was used to assess how EC altered between brain regions in DoC patients compared to healthy individuals.

**Results:**

We found increased effective connectivity in left striatum and decreased effective connectivity in bilateral precuneus (preCUN)/posterior cingulate cortex (PCC), bilateral midcingulate cortex and left middle frontal gyrus in DoC compared with the healthy controls. The resulting pattern of interaction in DoC indicated disrupted connection and disturbance of posterior parietal-frontal-striatum, and reduced self-inhibition of preCUN/PCC. The strength of self-inhibition of preCUN/PCC was negatively correlated with the total score of CRS-R.

**Conclusion:**

This impaired EC in DoC may underlie disruption in the posterior parietal-frontal-striatum circuit, particularly damage to the cortico-striatal connection and possible loss of preCUN/PCC function as the main regulatory hub.

## Introduction

Disorder of consciousness (DoC) includes several neurological conditions ranging from the minimally conscious state to unresponsive wakefulness syndrome or vegetative state, losing both responsiveness and consciousness, as a result of severe brain injuries. Resting-state functional magnetic resonance imaging (fMRI), describing spontaneous brain activity and generating intrinsic brain networks by calculating functional correlations between brain regions based on spontaneous blood oxygenation level dependent (BOLD) signal fluctuations, is one of the most employed techniques in the field of DoC. Several imaging studies have reported abnormality of crucial regions such as precuneus (preCUN)/posterior cingulate cortex (PCC), medial prefrontal cortex (mPFC), thalamus, and striatum related to consciousness dysfunction in DoC patients (Norton et al., 2012; Yao et al., 2015; Rosazza et al., 2016). Aberrant network connectivity has also been robustly and repeatably reported to be affected in DoC and several studies have reported that abnormal functional activity in the default mode network (DMN) is related to consciousness dysfunction and consciousness recovery in DoC patients (Vanhaudenhuyse et al., 2010; Rosazza et al., 2016; Amico et al., 2017). A common finding of studies on DoC is an impairment in the activity of high-order association cortices rather than “low-level” primary cortices.

However, as research continues, current theoretical views on consciousness have indicated widespread functional disconnection sufficient to lose awareness, rather than dysfunction in isolated regions (Dehaene et al., 2006; Seth et al., 2006). The deficits of cortico-subcortical (such as thalamus and striatum) and cortico-cortical connectivity have been recognized as the neuroanatomic basis of losing consciousness. The mesocircuit hypothesis was proposed to explain the vulnerability of the anterior forebrain (frontal/prefrontal cortical-striatopallidal thalamocortical loop systems; Schiff, 2010). Recent research of Adama et al. indicated the key functional role of the mediodorsal (MD) thalamus for the integrative function of the forebrain corticothalamic systems (Rikhye et al., 2012; Peräkylä et al., 2017; Schmitt et al., 2017; Parnaudeau et al., 2018). Monti et al. (2015) also reported impaired thalamo-frontal functional connectivity (FC) based on functional magnetic resonance imaging (fMRI) data acquired when DoC patients were performing a top-down cognitive task. Several other studies have found disrupted thalamocortical FC of DoC patients during resting-state (Tang et al., 2011; Zhou et al., 2011, 2014). Chen et al. (2018) estimated abnormal effective connectivity (EC) of the anterior forebrain regions which may be associated with patient prognosis. Major studies of cortico-cortical disconnection frame debates on the neural correlates of consciousness as “front versus back.” Arguments were mainly centered on contributions of frontal and posterior cortex in maintaining consciousness, with several theories proposed (Tononi et al., 2016; Farrell, 2018; Mashour et al., 2020). A growing body of studies has identified seed regions based on these theories (Tang et al., 2011; Zhou et al., 2011, 2014). Deficits in the frontal cortex, based on lateral fronto-parietal networks, are reported in DoC (Massimini et al., 2012; Crone et al., 2014). The posterior cortex, as DMN is based on midline fronto-parietal networks, is linearly related to the intensity of internal awareness. Silva et al. (2015) reported impaired functional connectivity between the midline preCUN/PCC and anterior cingulate/medial prefrontal cortices, considered as two core midline nodes of the DMN.

According to the research mentioned above, studies on global brain spontaneous activity using independent component analysis (Norton et al., 2012), amplitude of low-frequency fluctuation or FC strength (Wu et al., 2019) revealed altered brain intrinsic function within regions rather than interaction between regions. Abnormal effective connectivity of isolated brain regions discussed at circuit-level should be the goal of future studies. However, the above-mentioned fMRI research revealed abnormal functional connectivity in cortico-subcortical and cortico-cortical regions using a seed-based method based on *a priori* assumptions which may introduce bias, indicating a need for an unbiased strategy of selecting brain regions in further research.

To explore the crucial cortical and subcortical regions and verify contributions of these regions in DoC, we performed a meta-analysis on resting-state fMRI data extracted from an online database. Given that data are gathered from isolated brain regions using different methods, quantitative meta-analysis allows heterogeneous results of individual studies to be pooled and analyzed using a rigorous statistical framework that can identify regions of vulnerability associated with different levels of conscious awareness. Seed-based *d* mapping permutation of subject images (SDM-PSI) initially developed by Albajes-Eizagirre et al. (2019) is helpful to summarize the data of interest and provide brain regions of interest for further analysis. This voxel-based meta-analytic method allows a more exhaustive and unbiased inclusion of multiple voxel-based neuroimaging studies of normal brain functions and brain abnormalities in neuropsychiatric disorders, as well as more accurate estimations. Selecting the spatially extensive set of seed regions of interest (ROIs) provided in the meta-analysis, we aimed to organize the results into a coherent model of large-scale reciprocal connections between circuit-level regions.

The present study was designed to investigate the localization of the brain regions displaying altered activity in patients with DoC with a quantitative meta-analysis, summarizing the resting-state fMRI literature available to date. We compared EC among these regions and their directionality between DoC patients and healthy controls using spectral dynamic causal modeling (spDCM). Finally, we investigated the relationship between DoC and altered ECs between the regions.

## Materials and Methods

### Participants

In the current study, eye-closed and awake resting-state fMRI data were obtained in a total of 19 DoC patients and 17 age- and gender-matched healthy controls (HC). Patients’ diagnosis based on Coma Recovery-Scale-Revised (CRS-R) assessment (Giacino et al., 2004) was made by two specialists. Data from subjects with head motion more than 3 mm translation, 3° of rotation or large cerebral deformation were excluded from further analyses. Careful visual image quality inspection was used to exclude unsuccessful realignment and segmentation, and data from a total of 3 DoC patients and one control subject were excluded on this basis. Data from sixteen DoC patients (13M/3F, mean age 46 years, SD 15 years, 6MCS/10VS) and 16 demographically matched HCs (12M/4F, mean age 45 years, SD 16 years) were therefore included in further analysis. Demographic and clinical data of DoC patients and HCs are shown in Tables 1, 2. Informed consent was obtained directly from all healthy participants and the legal representative of all patients. The study was approved by the ethics committee of Wuhan University Zhongnan Hospital and was conducted in accordance with the tenets of the Declaration of Helsinki.

**TABLE 1:** Demographic characteristics of the patients with disorders of consciousness (DOC) and the healthy controls (HC).

Patient index	Gender	Age (years)	Time of fMRI (days after insult)	Etiology	CRS-R scores	MCS/VS
P01	M	75	15	Hypoxic brain injury	4	VS
P02	M	63	23	Hypoxic brain injury	7	VS
P03	F	26	48	Intracranial surgery	4	VS
P04	M	53	81	Hypoxic brain injury	7	VS
P05	M	75	592	Traumatic brain injury	9	MCS
P06	M	55	991	Intracerebral hemorrhage	9	MCS
P07	M	65	57	Intracerebral hemorrhage	4	VS
P08	M	63	172	Intracerebral hemorrhage	6	VS
P09	M	38	125	Traumatic brain injury	12	MCS
P10	F	48	96	Traumatic brain injury	6	VS
P11	F	26	165	Intracranial surgery	11	MCS
P12	M	32	310	Intracranial surgery	4	VS
P13	M	47	45	Hypoxic brain injury	7	VS
P14	M	73	64	Hypoxic brain injury	6	VS
P15	M	19	149	Hypoxic brain injury	9	MCS
P16	M	19	179	Hypoxic brain injury	11	MCS

**TABLE 2:** Demographic and clinical data of DoC patients.

Characteristics	DoC	HC	*P*-value
Gender (male/female)	13/3	12/4	*P* > 0.05
Age (years)	46 ± 15	45 ± 16	*P* > 0.05
Handedness (R/L)	15/1	16/0	*P* > 0.05
CRS-R scores	7.23 ± 2.67	–	–

*M, male; F, female.*

### Behavioral Assessment

The CRS-R score was acquired using largely preserved brainstem reflexes and preserved sleep–wake-cycle checked by neurological examination.

### MRI Data Acquisition

MRI data of all subjects were acquired on a 3.0 T Siemens Prisma scanner (Siemens AG, Healthcare Sector) using a 64-channel head coil. Each subject was required to keep in the supine position by a belt and foam pads during rest and awake condition with eyes closed. fMRI data was scanned using a gradient-echo echo-planer imaging (EPI) sequence of 240 volumes in an ascending interleaved order using the following protocols: repetition time (TR)/echo time (TE) = 2,000/40 ms, flip angle = 90°, field of view (FOV) = 240 mm × 240 mm, slice thickness = 4.0 mm, inplane resolution = 64 × 64, 32 axial slices with a slice gap of 1 mm. Then, high-resolution brain structural images were collected with a T1-weighted 3D magnetization-prepared rapid gradient-echo (MPRAGE) sequence (TR/TE = 1,900 ms/2.26 ms, matrix = 240 × 256, FOV = 215 mm × 230 mm, slice thickness/gap = 1.0/0 mm, 176 sagittal slices).

### Selection of Regions of Interest (ROI)

#### Search Strategy and Selection Criteria

**FIGURE 1:**
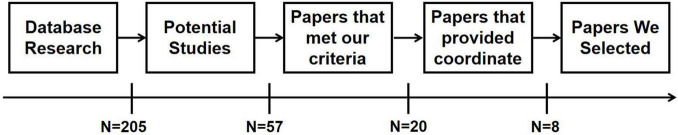
Flowchart of literature screening.

The literature search was conducted on whole-brain fMRI studies in DoC using the PubMed^1^ and Web of Science^2^ databases without publication date filter and combinations of the following keywords: “disorder of consciousness/DoC,” “unresponsive wakefulness syndrome,” “minimally conscious state,” “vegetative state” plus “resting-state fMRI” or “neuroimaging.” Due to the limited number of publications, we included any cause of DoC, including traumatic brain injury, anoxic brain injury, cardiac/cerebral vascular events, intoxication, and hypoxic ischemic encephalopathy, etc. After carefully screening the titles and abstracts of related studies, 57 potential studies were selected from 205 original studies for further scrutiny. Based on the rules for neuroimaging meta-analysis (Muller et al., 2018; Tahmasian et al., 2019), higher-quality criteria for study inclusion were as following: images covering the whole brain, inclusion of more than 10 subjects, whole-brain analyses, match for age and gender, use of standardized categorical or dimensional measures, definitive inclusion/exclusion criteria and description of software use and statistical methods. Studies were excluded if they were reviews, unrelated issues, without reporting the Montreal Neurological Institute (MNI)/Talairach coordinates (Lancaster et al., 2000) and T/Z value of clusters, ROI-based analysis, small-volume correction, studies of independent component analysis focused on only one resting-state network, or repeating data in two studies. Need of special note is that only studies in which DoC patients were combined rather than reporting separate information for each condition (e.g., VS and MCS) were included in the analysis. A total of 8 studies were finally selected for the following research (see Figure 1 and Table 3).

**TABLE 3:** Subject demographic in included studies.

Study	*N*	Analysis method	Gender (M/F)	Age in years (SD)	Space	Contrast
Huang et al., 2019	20	fALFF	13/7	53.30 (NA)	MNI	Doc<HC
Wu et al., 2019	15	FCS & fALFF	12/3	41.40 (13.22)	MNI	Doc<HC
He et al., 2014	12	fALFF	8/4	44.7 (17.5)	MNI	Doc<HC
Soddu et al., 2016	11	total neuronal activity	5/6	50 (14)	MNI	Doc<HC
Demertzi et al., 2014	53	ICA	33/20	50 (18)	MNI	Doc<HC
Huang et al., 2014	11	ALFF	10/1	44 (20)	MNI	Doc<HC
Yao et al., 2015	11	ALFF	6/5	43.1 (15.6)	MNI	Doc<HC
Salvato et al., 2020	14	fALFF	5/9	63.6 (8.5)	MNI	Doc<HC

*N, number; M, male; F, female; SD, Standard deviation; ReHo, Regional Homogeneity; fALFF, Fractional Amplitude of Low Frequency Fluctuations; FCS, functional connectivity strength; ALFF, Amplitude of Low Frequency Fluctuations; ICA, Independent Component Analysis; MNI, Montreal Neurological Institute; DoC, disorder of consciousness; HC, healthy control.*

#### SDM Meta-Analysis

Quantitative evaluation of meta-analysis was performed using the SDM software^3^ with MNI coordinates (Talairach coordinates were first converted into MNI coordinates) on the brain activation patterns for DoC and HC. The meta-analysis was performed in the following steps which have been described previously (Radua and Mataix-Cols, 2009; Radua et al., 2012). Firstly, an effect-size signed map and an effect-size variance map of given peak coordinates and their between-group *t*-values were extracted from individual studies using an anisotropic unnormalized Gaussian kernel with 20 mm full width at half maximum (FWHM) to control false-positive results. Then, the SDM generated a mean map by voxel-wise calculation of the mean of the dataset maps with a random-effect linear model, weighted by the sample size of each study, estimating intrastudy variability and inter-study heterogeneity. The result of heterogeneity analysis using a random-effects model with Q statistics represents between-study variability in the results. The above analyses were performed with a statistical significance of the following combination of thresholds: voxel probability threshold *p* < 0.005, peak height *Z* ≥ 1 and cluster extent threshold >10 voxels, achieving an optimal balance between sensitivity and specificity (Radua et al., 2012).

#### Functional Characterization of Identified SDM Meta-Analysis Result

To facilitate the functional interpretability of the identified clusters resulting from SDM meta-analysis, we used the Neurosynth database^4^ for data-driven characterization. Neurosynth is a database for large-scale, automated synthesis of functional magnetic resonance imaging (fMRI) data from >14,000 functional MRI studies and can be searched for the functional decoding of chosen clusters (Yarkoni et al., 2011). We selected the psychological terms from the association list with meta-analysis maps of the identified cluster in the Neurosynth database.

#### Acquisition and Preprocessing of Functional Magnetic Resonance Imaging Data

After converting the DICOM format of the functional data to NIFTI format, all preprocessing was completed using the Data Processing and Analysis of Brain Imaging (DPABI v4.0)^5^ toolbox (Yan et al., 2016) in a MATLAB (2013b, MathWorks Inc., Natick, MA, United States) platform. The standard preprocessing procedures based on the following steps: discarding the first ten volumes of each data set to avoid magnetization instability, slice-timing correction, head motion correction, spatial normalization to the Montreal Neurological Institute (MNI) space based on high-resolution T1WI registration, resampling to a voxel size of 3 × 3 × 3 mm^3^ and smoothed with a Gaussian kernel of 6 × 6 × 6 mm^3^ depending on the full width at half maximum (FWHM). Besides, the criterion of head motion was limited within 3 mm translations and 3° rotations.

#### Dynamic Casual Modeling

The spDCM analyses were performed using the DCM12 module in SPM12 (Wellcome Department of Cognitive Neurology, London, United Kingdom^6^). Based on results of meta-analysis, the averaged time series of each voxel in the left striatum, middle frontal gyrus (MFG), midcingulate cortex (MCC) and preCUN/PCC were extracted from each subject. The first eigenvectors were then extracted after the influences of head motion and low-frequency drift were removed using a generalized linear model (GLM) implemented in SPM12. A fully connected mode was specified meaning that bi-directional connections were identified between every pair of ROIs in each subject. No exogenous input was provided to this model of resting state fMRI data. For the given four ROIs, 2^4^ free parameters performing the effective connections among the ROIs. Spectral DCM obtains a prediction of data feature based on the Fourier transform of the cross-correlation of the time series, previously described in detail (Yan et al., 2016).

To determine the best fitting model for each group through all possible dynamic causal models, a *post hoc* model selection routine fitting the full model of all free parameters was applied (Friston and Penny, 2011). As there are 2^4^ free parameters in the current estimation, a “greedy search” was efficiently implemented to result in 2^8^ = 256 reduced models (Rosa et al., 2012). A *post hoc* model optimization routine was then used to determine the best fitting model for each group based on posterior probability. The fully connected model has the highest evidence among distribution of log probability over the 256 reduced models. After the optimized model was selected, one sample *T*-test and two sample *T*-test were performed to compare the parameter inference of endogenous connections (DCM.Ep.A) within and between the groups.

**FIGURE 2:**
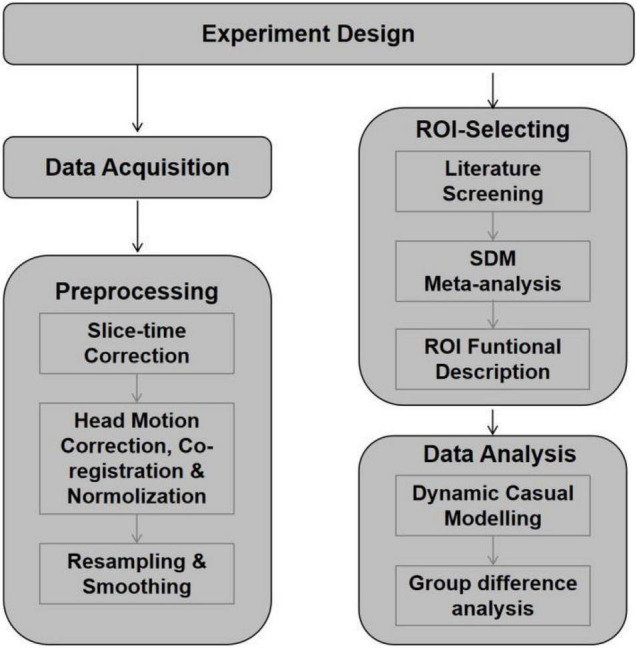
Flow diagram shows the experiment designing of the current study.

Figure 2 shows the experiment designing of the current study.

## Results

### Result of Meta-Analyses

**FIGURE 3:**
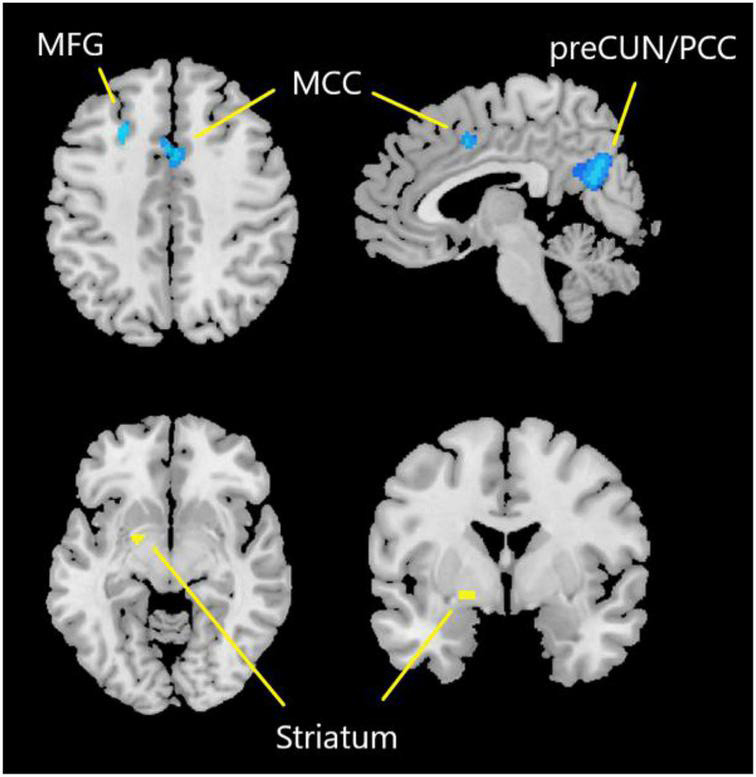
Results of SDM-PSI meta-analysis. Yellow represents the brain region with increased activity. Blue represents the brain region with decreased activity. preCUN, precuneus; PCC, posterior cingulate cortex; MCC, middle cingulate cortex; MFG, middle frontal gyrus.

One hundred and forty-seven subjects with DoC (92 males) were included from eight studies, details of which are shown in Table 3. Figure 3 and Table 4 show the SDM of altered activity in patients with DoC at resting-state compared to healthy subjects. Result on the entire sample of DoC patients was constituted of 4 separate regions highlighting the involvement of deactivated cortical areas (bilateral preCUN/PCC, left MCC, bilateral MFG) and activation in left striatum.

**TABLE 4:** Results of meta-analysis were selected for following spDCM analyses.

Regions	Hemisphere	MNI coordinates	SDM-Z value	*P* value	voxel
Striatum	L	−20, 0, −6	6.142	<0.05	16
PreCUN/PCC	B	−2, −66, 26	−8.057	<0.0001	594
MCC	B	2, 8, 44	−6.529	<0.001	131
MFG	L	−26, 18, 48	−6.658	<0.005	43

*MNI, Montreal Neurological Institute; L, left; R, right; preCUN, precuneus; PCC, posterior cingulate cortex; MCC, midcingulate cortex; MFG, middle frontal gyrus.*

### Functional Characterization of the Identified Clusters

**FIGURE 4:**
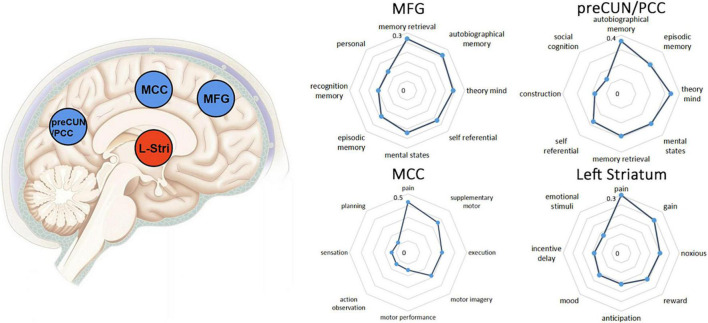
The left column showed brain regions showing positive (blue) or negative (yellow) correlation with DoC in the meta-analysis. Clusters were displayed at voxel probability threshold *P* < 0.005, peak height *Z* ≥ 1 and cluster extent threshold >10 voxels. The right column showed functional characterization of identified clusters. The statistics in the radar charts are *r* values from Pearson’s correlations. In the current Neurosynth framework, the *r* values reflect the correlation between two maps. preCUN, precuneus; PCC, posterior cingulate cortex; MCC, middle cingulate cortex; MFG, middle frontal gyrus.

Figure 4 depicts the functional profiles of preCUN/PCC, MFG, MCC and left striatum defined by NeuroSynth. Several highly correlated terms with similar functional meanings were merged, for example, memory retrieval was the combination of memory retrieval and retrieval. The exact *r* values of extracted psychological terms mean value of Pearson correlation between functional terms and the selected brain region.

### Bayesian Model Selection and Dynamic Causal Modeling

**FIGURE 5:**
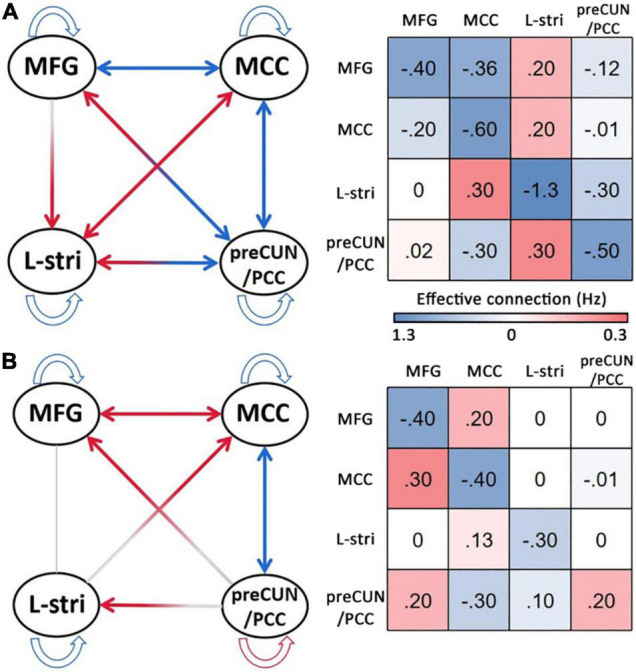
The result of DCM of HC **(A)** and DoC **(B)**. L, left; R, right; Stria, striatum; preCUN, precuneus; PCC, posterior cingulate cortex; MCC, middle cingulate cortex; MFG, middle frontal gyrus.

**FIGURE 6:**
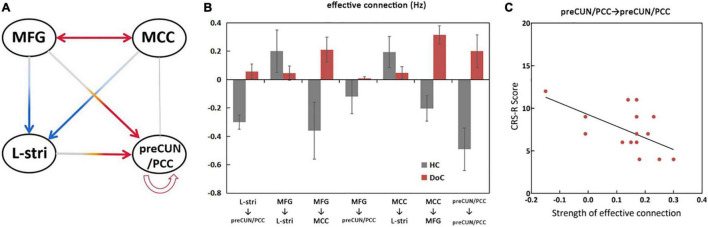
Significant changes in effective connections (Hz) in the DOC patients. Red arrows, connections with significant increases; blue arrows, connections with significant decreases (*P* < 0.05) **(A)**. Mean effective connections of significantly changed pairs of regions **(B)**. The strength of self-connection of preCUN/PCC is negatively correlated with the score of CRS-R **(C)**. L, left; R, right; Stria, striatum; preCUN, precuneus; PCC, posterior cingulate cortex; MCC, middle cingulate cortex; MFG, middle frontal gyrus.

The significant differences in effective connectivity strength between the DoC patients and controls are shown in Figures 5, 6. Strength of positive connectivity between both MFG and MCC and the left striatum was significantly lower in DoC patients than controls (*P* < 0.05). The strength of the connection between the left striatum and preCUN/PCC in HCs was not reported in DoC (while reported as increased mathematically) (*P* < 0.05). We observed that the strength of negative connectivity between MFG and MCC, disappeared negative connectivity strength between MFG and preCUN/PCC and self-connection of preCUN/PCC was observed as positive in DoC patients (*P* < 0.05).

To further explore the relationship between clinical scale and properties of effective connectivity in patients, Pearson’s correlation was calculated between the CRS-R scores and those values, showing statistically significant differences between the two groups. The strength of self-connection of preCUN/PCC was negatively correlated with the CRS-R score in DoC patients (*P* < 0.05) (Figure 6C).

## Discussion

Our meta-analysis results show diminished activity in preCUN/PCC, MCC and MFG (higher order areas of cognition) in DoC, in agreement with previous research (Laureys, 2005). In DCM analysis of resting-state fMRI, values of connectivity strength made sense of rate constants of neuronal responses, interpreted as reciprocal connectivity. We identified the specific ECs and directionality among these regions at resting-state in DoC patients compared with controls. In particular, our results of the functional integrity of cortical-striatum and posterior parietal-frontal connectivity indicated the abnormal functional connectivity between network of intrinsic awareness and extrinsic awareness. Importantly, the self-inhibition of preCUN/PCC appeared to be related to the degree of consciousness impairment.

### Differences in the Cortical-Cortical Connectivity Between the Two Groups

PreCUN/PCC, as a component of the DMN, is considered crucial to maintain consciousness. At resting state, preCUN/PCC plays the role of a central and structural hub of information input from multiple brain regions (Hagmann et al., 2008; Deshpande et al., 2011). Previous studies of effective connectivity using DCM of resting-state fMRI in healthy individuals have highlighted the role of the posterior cingulate cortex (PCC) as a main connector hub between distinct networks in healthy individuals (Hagmann et al., 2008; van den Heuvel and Sporns, 2011; Li et al., 2012). We demonstrated that the disruption of self-inhibition and neuronal oscillations in preCUN/PCC is negatively correlated with the total score of CRS-R. This finding is generally consistent with previous research (Crone et al., 2015). The long afferent and efferent axonal fibers of preCUN/PCC connecting to distant cortical and subcortical targets, as well as the high resting state metabolic activity in this region make it vulnerable to damage (Hannawi et al., 2015). Afrasiabi et al. (2021) showed the contribution of parietal cortex in detecting changes in consciousness and in the integration of cortico-striatal-thalamic activity. Our results suggest that preCUN/PCC functions as the regulatory hub among cortical regions maintaining equilibrium between excitatory and inhibitory connectivity may be lost in DoC patients.

The regions MFG and MCC play a role in advanced cognitive functions such as aspects of memory, mental state and self-reference (Figure 1; Jin et al., 2018). Our results indicated that DoC patients have disrupted connectivity between the posterior parietal (preCUN/PCC) and frontal (MFG) cortex, related to two distinct and negatively correlated networks of consciousness. It has been proposed that loss of consciousness may be associated with a deterioration of the functional dynamics between mid-line regions of the frontal and parietal cortices. Evidence has accumulated from studies showing widespread disconnection between frontal and parietal regions in patients with impaired consciousness (Boly et al., 2009; Threlkeld et al., 2018). Laureys and Schiff proposed a model of recovery of consciousness which emphasized the contribution of network connectivity between frontal and parietal regions (Laureys and Schiff, 2012). Bonini et al. (2016) found changes in synchrony in frontal and parietal regions associated with loss of consciousness in frontal lobe epilepsy. Similar breakdowns in intracortical connectivity of frontal regions (ventral attention network) and parietal regions (DMN) have also been identified in sevoflurane-induced unconsciousness (Palanca et al., 2017). Our current result also indicated the altered effective function connectivity from MFG to preCUN/PCC may related with maintenance of consciousness.

### Impaired Effective Connections of Corticostriatal Circuitry in Disorder of Consciousness Patients

The central role of the striatum in consciousness and the necessary link between the disrupted corticostriatal connections and loss of consciousness are highlighted by the present results. Previous studies consider the striatum to be an important subcortical structure with several motor and cognitive functions (Liu et al., 2020) rather than a brain area contributing to consciousness (Liu et al., 2020). However, a growing body of literature has suggested altered striatum activity in hallucinogen-induced altered states of consciousness (Slagter et al., 2017), general anesthesia (Mhuircheartaigh et al., 2010) and the sleep-wake cycle (Braun et al., 1997). The striatum also contributes to consciousness through reciprocal connections with the thalamus and cortical regions according to the mesocircuit theory (Schiff, 2010). Lacey et al. (2007) indicated a sharply reduced output of medium spiny neurons in the striatum of diffuse brain injury subjects which lead to a reduction of direct excitatory input from the central thalamus and down-regulation of corticostriatal input (Schiff, 2010). These existing models have explained the mechanism of impaired metabolic activity of the striatum during changing states of consciousness.

In support of the complex and extensive corticostriatal circuitry theory (Haber, 2016), we found reduced MFG-striatum, MCC-striatum and preCUN/PCC-striatum connectivity in DoC patients compared with controls. As the main source of input to the basal ganglia, the striatum is connected with the cerebral cortex through the ganglia-cortical loop (Albin et al., 1989). In healthy volunteers, DCM has demonstrated a negative effective connection between cortical nodes with preCUN/PCC as the main hub (Soch et al., 2017), with the striatum receiving excitatory input from all other cortical nodes. Both input and output of cortical regions show a trend toward reduced or absent activity in DoC. Several current models indicate that the direct and indirect efferent pathways from the striatum, influencing basal ganglia output nuclei, play important roles in the regulation of thalamocortical and brainstem motor circuits (Smith et al., 1998; Kravitz et al., 2010; Freeze et al., 2013), thought to be fundamental to basic motor functions of survival. The frontal-striatal circuit is also well described in healthy populations, implicated in behavioral alterations in diseases of motor dysfunction (Quan et al., 2013; Chen et al., 2018). Alexander et al found reduced or absent effective connectivity between cortex and striatum in anesthetized rats compared with free behavior rats (Nakhnikian et al., 2014). Striatal activity is also observed to be strongly reduced when epilepsy patients lose consciousness (David et al., 2008; Moeller et al., 2008). Taking these studies together, the disappeared MFG-striatum may explain movement disorders in DoC patients. However, Nicholas et al reported disrupted structural integrity of preCUN and bilateral striatum in DoC patients, perhaps suggesting a loss of striatal output and dysregulation between the preCUN and the striatum (David et al., 2008; Moeller et al., 2008). Another DCM study by Crone et al. (2017) indicated a breakdown of basal ganglia-cortical (including PCC) connectivity in a loss of consciousness. Considering the sensory inputs that the striatum receives from multiple cortical sources, our result likely reflects the necessity of integrated corticostriatal circuitry to maintain consciousness.

## Limitations

The present study has some limitations. Firstly, we included any cause of DoC when recruiting patients and in the meta-analysis. We excluded some patients before data acquisition and discarded several others based on relatively strict procedures of quality control, reducing the sample size. A larger cohort is needed to study the different types of DoC caused by different etiologies and this will be a focus in our future work in this area. Secondly, our study has limitations inherent to meta-analyses. The meta-analysis study design involves data extracted from published studies rather than raw data and statistics, increasing the variance in the pooled data and possibly reducing the accuracy of results. Thirdly, although a number of DOC patients were recruited for data acquisition, we were only able to utilize 16 patients for the analysis. There was an agonizing trade-off between subject quantity and image quality. The extensive brain lesion in some patients would have produced poor normalization results and overlarge head-motions would have introduced artifacts. Because of these reasons, the sample size of this study is small and it is difficult to divide DoC patients into MCS and VS again, which is also what we need to make efforts in the future.

## Conclusion

Using meta-analysis, we identified decreased activity of preCUN/PCC, MFG and MCC and increased activity of the left striatum in DoC patients. The resulting pattern of interaction in DoC identified disrupted connection, disturbance of the posterior parietal-frontal-striatum circuit, and reduced self-inhibition of preCUN/PCC. These impairments may be principally due to the disruption of mechanisms underlying damage of corticostriatal connection and possible loss of function of preCUN/PCC, a significant regulatory hub.

## Data Availability Statement

The raw data supporting the conclusions of this article will be made available by the authors, without undue reservation.

## Ethics Statement

The studies involving human participants were reviewed and approved by the Ethics Committee of Zhongnan Hospital of Wuhan University. The participants and family member of patients provided their written informed consent to participate in this study.

## Author Contributions

All authors listed have made a substantial, direct, and intellectual contribution to the work, and approved it for publication.

## Conflict of Interest

The authors declare that the research was conducted in the absence of any commercial or financial relationships that could be construed as a potential conflict of interest.

## Publisher’s Note

All claims expressed in this article are solely those of the authors and do not necessarily represent those of their affiliated organizations, or those of the publisher, the editors and the reviewers. Any product that may be evaluated in this article, or claim that may be made by its manufacturer, is not guaranteed or endorsed by the publisher.

## Footnotes

1
https://pubmed.ncbi.nlm.nih.gov/

2
https://www.webofknowledge.com/

3
https://www.sdmproject.com/

4
http://www.neurosynth.org

5
http://www.rfmri.org/dpabi

6
http://www.fil.ion.ucl.ac.uk/spm/
